# Association between follicle‐stimulating hormone and nonalcoholic fatty liver disease in postmenopausal women with type 2 diabetes mellitus

**DOI:** 10.1111/1753-0407.13394

**Published:** 2023-05-23

**Authors:** Shengjie Ge, Yinfeng Zheng, Linjia Du, Xiang Hu, Jingzong Zhou, Zhiying He, Xiao Gu, Xiaoyan Huang, Lijuan Yang, Xiuli Lin, Xuejiang Gu

**Affiliations:** ^1^ Department of Endocrine and Metabolic Diseases The First Affiliated Hospital of Wenzhou Medical University Wenzhou China; ^2^ Department of Infectious Diseases The First Affiliated Hospital of Wenzhou Medical University Wenzhou China

**Keywords:** follicle‐stimulating hormone, nonalcoholic fatty liver disease, obesity, postmenopausal women, 卵泡刺激素, 非酒精性脂肪性肝病, 肥胖, 绝经后妇女

## Abstract

**Background and Aim:**

Follicle‐stimulating hormone (FSH) was negatively associated with nonalcoholic fatty liver disease (NAFLD) in women older than 55 years old. People with obesity and diabetes had higher prevalence of NAFLD. Thus, we aimed to explore the association between FSH and NAFLD in postmenopausal women with type 2 diabetes mellitus (T2DM).

**Methods:**

A total of 583 postmenopausal women with T2DM with an average age of 60.22 ± 6.49 were recruited in this cross‐sectional study through January 2017 to May 2021. Anthropological data, biochemical indexes, and abdominal ultrasound results were retrospectively collected. Abdominal ultrasound was used to diagnose NAFLD. FSH was measured by enzymatic immunochemiluminescence and divided into tertiles for further analysis. The logistic regression was used to assess the association of FSH with prevalent NAFLD. Likelihood ratio tests were used to assess the interactions between groups.

**Results:**

A total of 332 (56.94%) postmenopausal women had NAFLD. Compared with postmenopausal women in the lowest tertile of FSH, postmenopausal women in the highest tertile of FSH had lower prevalence of NAFLD (*p* < .01). After adjusting for age, diabetes duration, metabolism‐related indicators, and other sex‐related hormones, FSH was inversely associated with NAFLD (odds ratio: 0.411, 95% confidence intervals: 0.260–0.651, *p* < .001). In subgroup analysis, there were no significant interactions of FSH with strata of metabolic factors on the association of NAFLD.

**Conclusion:**

FSH was negatively and independently associated with NAFLD in postmenopausal women with type 2 diabetes mellitus. It might be a potential index for screening and identifying individuals with high risk of NAFLD in postmenopausal women.

## Graphical abstract

**Highlights**

- In Chinese postmenopausal women with type 2 diabetes mellitus, serum follicle‐stimulating hormone levels were negatively and independently associated with the prevalence of nonalcoholic fatty liver disease.
- Metabolic conditions, including overweight/obesity, central obesity, hyperuricemia, hypertension, and dyslipidemia did not interact with this association.
- Follicle‐stimulating hormone might be a potential independent index for screening and identifying individuals with high risk of non‐alcoholic fatty liver disease in postmenopausal women with type 2 diabetes mellitus.

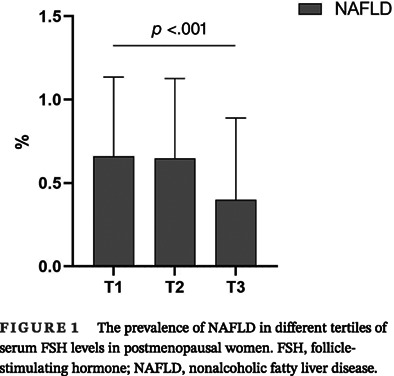

## 摘要

背景:卵泡刺激素(FSH)与55岁以上女性非酒精性脂肪性肝病(NAFLD)呈负相关。肥胖和糖尿病人群NAFLD患病率较高。因此, 本研究旨在探讨绝经后2型糖尿病(T2DM)女性中FSH与NAFLD的关系。

方法:本研究为横断面研究, 于2017年1月至2021年5月招募583例绝经后女性T2DM患者, 平均年龄(60.22±6.49)岁。回顾性收集患者的人体测量数据、生化指标及腹部超声检查结果, 并应用腹部超声诊断NAFLD。采用酶免疫化学发光法测定FSH, 并分为三分位数进行分析。采用logistic回归分析FSH与NAFLD患病率的关系。采用似然比检验评估组间交互作用。

结果:共332例(56.94%)绝经后女性患NAFLD。与FSH水平最低三分位的绝经后女性相比, FSH水平最高三分位的绝经后女性NAFLD患病率较低(P<0.01)。校正年龄、糖尿病病程、代谢相关指标和其他相关性激素后, FSH与NAFLD呈负相关(优势比(OR): 0.411, 95%可信区间(CI): 0.260 ~ 0.651, P<0.001)。在亚组分析中, FSH与各代谢因素的交互作用对NAFLD的影响无统计学意义。

结论:在绝经后2型糖尿病患者中, FSH与NAFLD呈独立负相关, 可作为绝经后女性筛查和识别NAFLD高危人群的潜在指标。

## 1 INTRODUCTION

Nonalcoholic fatty liver disease (NAFLD) is a metabolism‐related liver disease, accompanied by steatosis in more than 5% of liver cells.^1^, ^2^ At present, NAFLD has become the most common chronic liver disease in the world, with a global prevalence of about 25%.^3^ In China, the prevalence of NAFLD is as high as 29.2%, and its prevalence is still increasing, which has become a major public health problem that cannot be ignoref.^4^ NAFLD is associated with obesity, type 2 diabetes mellitus (T2DM), hypertension, dyslipidemia, and insulin resistance.^5^, ^6^, ^7^ Among these risk factors, T2DM has the greatest impact on NAFLD.

As a metabolic disease, T2DM and its complications greatly increase the burden of mortality and disability worldwide. In China, the estimated age‐adjusted prevalence of T2DM is 10.6%, about 145 million people.^8^ T2DM is associated with NAFLD; around 70% of patients with T2DM have NAFLD.^9^ In addition, a meta‐analysis showed that T2DM can increase the risk of severe liver disease by more than two times.^10^ Therefore, for patients with T2DM, early identification and intervention of NAFLD to present its further progression are crucial.

Follicle‐stimulating hormone (FSH) is a glycoprotein peptide hormone produced by the anterior lobe of the pituitary gland.^11^ It affects both reproductive system and nonreproductive systems, such as osteoclasts^12^ and cholangiocytes.^13^ In 2004, Dhindsa et al first reported hypogonadotropin in males with T2DM, who had significant reductions in LH and FSH levels.^14^ In recent years, studies have found serum FSH level was negatively associated with diabetes, obesity, and metabolic syndrome in postmenopausal women.^15^, ^16^, ^17^ Wang et al found that serum FSH level was negatively associated with NAFLD in Chinese women over 55 years, which was mainly related to obesity and insulin resistance.^18^ However, the association between FSH and NAFLD in postmenopausal women with T2DM has not been studied.

Therefore, this study aimed to explore the association between FSH and NAFLD in postmenopausal women with T2DM and whether this association changed under different metabolic conditions.

## 2 METHODS

### 2.1 Subjects

This cross‐sectional study recruited 726 postmenopausal women with diabetes, aged between 41 and 80 years, through January 2017 to May 2021 in the department of endocrine and metabolic diseases, the First Affiliated Hospital of Wenzhou Medical University. The inclusion criteria were as follows: (a) females with menopause history, which was defined as having stopped menstruating for a minimum of 12 months without having a hysterectomy or oophorectomy; (b) meet the diagnostic criteria for T2DM; and (c) patients voluntarily received questionnaires, physical examinations, and laboratory tests. Patients were excluded based on the following criteria: (a) using hormone replacement therapy during 3 months before the baseline investigation; (b) having a history of excessive drinking (≥30 g/d for men and ≥20 g/d for women); (c) viral hepatitis and other hepatic disease; (d) other disease that may influence the levels of FSH; (e) type 1 diabetes mellitus and other special types of diabetes; (f) severe liver and kidney dysfunction; and (g) lack of FSH level or abdominal ultrasound results. Finally, 583 postmenopausal women were enrolled in the final analysis. This study had been approved by the Ethics Committee of the First Affiliated Hospital of Wenzhou Medical University. Informed consent was obtained from all patients for inclusion in this study.

### 2.2 Clinical and laboratory evaluation

Anthropometric data such as age, height, weight, waist circumference (WC), hip circumference (HC), blood pressure (BP), diabetes duration, drinking history, and menopausal status were collected at the time of sample collection. WC was measured to the nearest 0.1 cm at the midpoint between the margin of the last rib and the iliac crest of the ilium. HC was measured at the widest part of the pelvic region. Body mass index (BMI) was calculated according to the formula: BMI = weight (kg)/height^2^ (m^2^). Insulin resistance was estimated by the homeostatic model assessment‐insulin resistance(HOMA‐IR) index: (fasting insulin [mIU/L]) × (fasting plasma glucose [FPG] [mmol/L])/22.5.

Biochemical measurements were performed after fasting for at least 8 h. Glycosylated hemoglobin A1c (HbA1c) was tested using affinity chormatography (Hb9210, China). Luteinizing hormone (LH), FSH, prolactin, estradiol, progesterone, testosterone, and fasting insulin level were determined by enzymatic immunochemiluminescence (Beckman DxI 800, USA). The automatic biochemical instrument detection method (Beckman AU5800, USA) was used to determine the levels of FPG, triglycerides, total cholesterol, high‐density lipoprotein cholesterol, low‐density lipoprotein cholesterol, uric acid, alanine aminotransferase, and aspartate aminotransferase.

Diagnosis of NAFLD was based on the abdominal ultrasound.^1^ As a noninvasive, widely used, and accurate tool, abdominal ultrasound was performed by two experienced radiologists. On ultrasound, NAFLD was characterized by bright hepatic echo, enhanced hepatorenal echogenicity, and blurring of portal or hepatic vein vessels.^19^

### 2.3 Definition of variables

Diabetes was defined as FPG ≥7.0 mmol/L or 2‐h plasma glucose ≥11.1 mmol/L during steamed bran meal experiment or HbA1c ≥6.5% or a random plasma glucose ≥11.1 mmol/L with typical hyperglycemia symptoms or treatment with a hypoglycemic agent or insulin. Good glycemic control was defined as HbA1c < 7.0%.^20^ In the Chinese population, BMI ≥24 kg/m^2^ was considered as overweight^21^ and WC ≥85 cm in women was considered as central obesity.^22^ Hypertension was defined as systolic BP ≥140 mm Hg and/or diastolic BP ≥90 mm Hg without using antihypertensive medication or BP < 140/90 mm Hg but under antihypertension treatment.^23^ Dyslipidemia was defined as triglycerides ≥2.3 mmol/L or total cholesterol ≥6.2 mmol/L or high‐density lipoprotein cholesterol <1.0 mmol/L or low‐density lipoprotein cholesterol ≥4.1 mmol/Lor the patient was receiving lipid‐lowering drugs.^22^ FSH was divided into tertiles (T1: FSH ≤52.92 IU/L; T2: 52.92 < FSH ≤72.55 IU/L; T3: FSH > 72.55 IU/L).

### 2.4 Statistical analyses

All statistical analyses were carried out using SPSS software, version 25.0 (SPSS Inc, Chicago, IL). The continuous variable was expressed as the mean ± SD or median, with the interquartile range according to a normal or skewed distribution, respectively. The categorical variables are expressed as percentages. Baseline characteristics were compared using one‐way analysis of variance analysis for normal continuous variables, nonparametric rank‐sum test for skewed continuous variables, and chi‐square test for categorical variables. The association of FSH with NAFLD was assessed by multivariate logistics regression analysis. Pearson correlation analysis was used to analysis the correlation between FSH and metabolic factors. Likelihood ratio tests were used assessed the interactions between groups. Model 1: unadjusted; Model 2: adjusted for age, BMI, WC, diabetes duration, hypertension; Model 3: adjusted for age, BMI, WC, diabetes duration, hypertension, hyperuricemia, blood glucose control, dyslipidemia, LH, prolactin, estradiol. *p* was calculated by multivariate logistic regression analyses. *p* value <.05 was considered statistically significant.

## 3 RESULTS

### 3.1 Clinical characteristics of the study population

**FIGURE 1:**
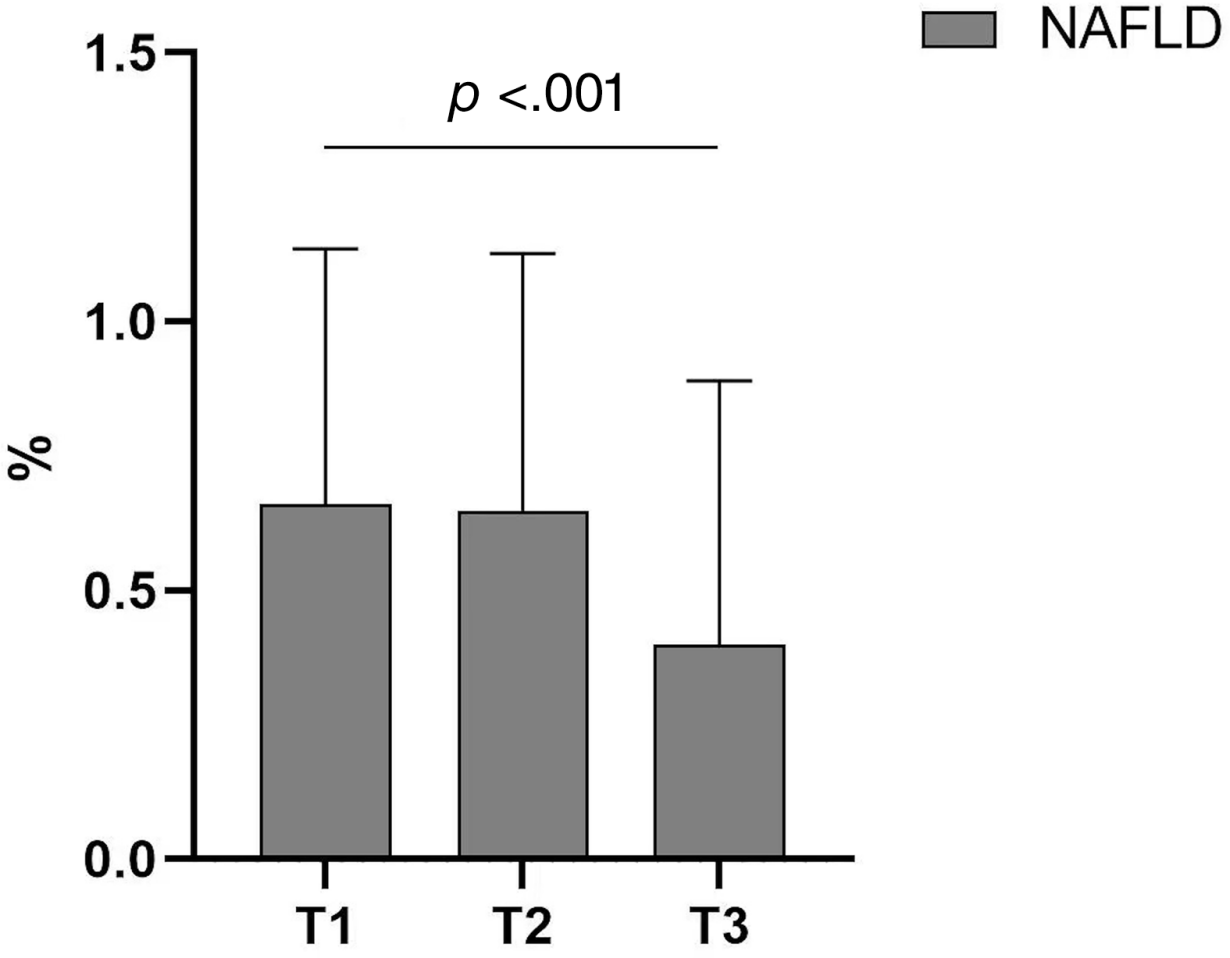
The prevalence of NAFLD in different tertiles of serum FSH levels in postmenopausal women. FSH, follicle‐stimulating hormone; NAFLD, nonalcoholic fatty liver disease.

The clinical characteristics of the study population with different tertiles of FSH were shown in Table 1. Among the 583 participants, the prevalence of NAFLD was 56.94%. With the increase of serum FSH level, the prevalence of NAFLD decreased (Figure 1). In addition, in Table 1, compared with postmenopausal women in the lowest tertile of FSH, postmenopausal women in the highest tertile of FSH had lower BMI, WC, and HC and higher serum LH and prolactin levels. There was no significant difference between age, BP, diabetes duration, FPG, HbA1c, liver function, uric acid, blood lipids, estradiol, progesterone, and testosterone.

**TABLE 1:** Clinical characteristics of the study population.

	T1	T2	T3	*p*
FSH (IU/L)	≤52.92	52.92–72.55	>72.55	
NAFLD/N (%)	128/194 (65.98%)	127/196 (64.80%)	77/193 (39.90%)	<.001
Age (y)	59.82 ± 6.79	60.63 ± 6.14	60.22 ± 6.54	.475
Systolic BP (mm Hg)	133.84 ± 20.79	131.73 ± 20.11	132.46 ± 20.54	.587
Diastolic BP (mm Hg)	76.27 ± 11.69	74.47 ± 12.63	73.48 ± 11.94	.072
Diabetes duration (m)	114 (46.50–179.50)	127 (56–190)	120 (56–180)	.410
Anthropometric data				
BMI (kg/m2)	25.22 ± 3.70	24.02 ± 3.04	23.20 ± 3.25	<.001
WC (cm)	90.75 ± 10.17	88.02 ± 8.72	85.24 ± 9.04	<.001
HC (cm)	94.69 ± 7.87	93.48 ± 6.77	92.04 ± 6.90	.002
Biochemical Measurements				
HbA1c (%)	9.82 ± 2.26	9.70 ± 2.18	9.58 ± 2.32	.579
FPG (mmol/L)	9.06 ± 2.71	9.12 ± 2.76	9.02 ± 2.78	.941
HOMA‐IR	3.23 (1.99–5.81)	2.92 (1.76–4.79)	2.45 (1.55–4.46)	.456
ALT (U/L)	20 (14–32.5)	19 (14–27)	19 (13–30)	.143
AST (U/L)	22 (17–31)	20 (17–25)	22 (17–29.5)	.069
UA (μmol/L)	289 (243.50–354)	278 (239–342)	297 (230.50–364.50)	.644
TG (mmol/L)	1.57 (1.15–2.27)	1.57 (1.11–2.18)	1.41 (1.03–2.14)	.144
TC (mmol/L)	4.93 (4.08–5.88)	4.92 (3.91–5.86)	4.81 (3.77–6.00)	.853
HDL‐c (mmol/L)	1.07 (0.93–1.22)	1.10 (0.93–1.25)	1.07 (0.91–1.33)	.705
LDL‐c (mmol/L)	2.74 (2.00–3.37)	2.43 (1.96–3.38)	2.43 (1.79–3.40)	.448
Sex hormones				
LH (IU/L)	17.42 (14.06–22.38)	24.67 (21.07–29.04)	35.74 (28.12–44.86)	<.001
PRL (μg/L)	6.91 (4.72–9.60)	6.21 (4.99–8.86)	7.86 (5.78–11.31)	<.001
Estradiol (pmol/L)	102.20 (73.00–154.30)	98.50 (73.00–139.80)	101.40 (73.00–129.15)	.175
Progesterone (nmol/L)	0.59 (0.25–1.24)	0.57 (0.27–0.95)	0.65 (0.39–1.21)	.226
Testosterone (nmol/L)	0.67 (0.36–1.19)	0.81 (0.39–1.21)	0.80 (0.40–1.20)	.736

*Note*: Data are summarized as the mean ± SD or median (interquartile range) for normally or nonnormally distributed continuous variables and as number with proportion for categorical variables. The one‐way analysis of variance analysis and nonparametric rank‐sum test were used for continuous variables with a skewed or normal distribution, and the Pearson χ2‐test was used for categorical variables.

Abbreviations: ALT, alanine aminotransferase; AST, aspartate aminotransferase; BMI, body mass index; BP, blood pressure; FPG, fasting plasma glucose; FSH, follicle‐stimulating hormone; HbA1c, glycosylated hemoglobin A1c; HC, hip circumference; HDL‐c, high‐density lipoprotein cholesterol; HOMA‐IR, homeostasis model assessment of insulin resistance; LDL‐c, low‐density lipoprotein cholesterol; LH, luteinizing hormone; NAFLD, nonalcoholic fatty liver disease; PRL, prolactin; TC, total cholesterol; TG, triglycerides; UA, uric acid; WC, waist circumference.

### 3.2 Association between the FSH and NAFLD

The association between the serum FSH level and NAFLD was presented in Table 2 by using multivariate logistic analyses. In an unadjusted model, the odds ratio (OR) of NAFLD was 0.342 (95% confidence interval [CI]: 0.226–0.518, *p* < .001) in the highest tertile of FSH, compared with the lowest tertile of FSH. After adjusting for age, general obesity, abdominal obesity, diabetes duration, hypertension, glycemic control, dyslipidemia, hyperuricemia, LH, PRL, and estradiol, the negative association between the serum FSH level and NAFLD still remained significant (OR: 0.411, 95% CI: 0.260–0.651, *p* < .001).

**TABLE 2:** Odds ratios with 95% confidence intervals for NAFLD according to tertiles of FSH in all subjects.

	Case/participants	Model 1		Model 2		Model 3	
		OR (95% CI)	*p*	OR (95% CI)	*p*	OR (95% CI)	*p*
T1	128/194	Reference		Reference		Reference	
T2	127/196	0.949 (0.625–1.441)	.806	1.152 (0.733–1.813)	.539	1.179 (0.742–1.874)	.486
T3	77/193	0.342 (0.226–0.518)	<.001	0.412 (0.264–0.645)	<.001	0.411 (0.260–0.651)	<.001

*Note*: Model 1: unadjusted. Model 2: adjusted for age, body mass index, waist circumference, diabetes duration, hypertension. Model 3: adjusted for age, body mass index, waist circumference, diabetes duration, hypertension, hyperuricemia, blood glucose control, dyslipidemia, luteinizing hormone, prolactin, estradiol. *P* was calculated by multivariate logistic regression analyses.

Abbreviations: CI, confidence intervals; FSH, follicle‐stimulating hormone; NAFLA, nonalcoholic fatty liver disease; OR, odds ratio.

#### 3.2.1 Correlation of FSH with metabolic factors

Table 3 showed the results of the Pearson correlation analysis between FSH and metabolic factors in postmenopausal women. FSH were negatively correlated with BMI, WC, and HOMA‐IR (*p* < .05).

**TABLE 3:** Pearson correlation of FSH with metabolic factors in postmenopausal women.

Variable	*r*	*p*
Age	0.016	.693
BMI	−0.238	<.001
WC	−0.235	<.001
HbA1c	−0.052	.208
lg‐TG	−0.047	.258
lg‐TC	−0.010	.814
lg‐HDL‐c	−0.060	.150
lg‐LDL‐c	−0.049	.240
lg‐HOMA‐IR	−0.094	.033

*Note*: Because TG, TC, LDL‐c, HDL‐c, and HOMA‐IR were skewed distribution, they were log transformed.

Abbreviations: BMI, body mass index; FSH, follicle‐stimulating hormone; HDL‐c, high‐density lipoprotein cholesterol; HOMA‐IR, homeostasis model assessment of insulin resistance; LDL‐c, low‐density lipoprotein cholesterol; TC, total cholesterol; TG, triglycerides; WC, waist circumference.

#### 3.2.2 Subgroup analysis

To further explore the relationship between the serum FSH level and NAFLD, interaction analysis was used to analyze the association between serum FSH level and NAFLD in subgroups, including age, diabetes duration, overweight and obesity, central obesity, hyperuricemia, hypertension, and dyslipidemia.

As showed in Table 4, age, diabetes duration, overweight/obesity, central obesity, hyperuricemia, hypertension, and dyslipidemia did not interact with FSH in relation to NAFLD (*p* for interaction was .571, .261, .922, .062, .568, .165, .414, respectively).

**TABLE 4:** Subgroups analyses for prevalent NAFLD among 583 participants with the top vs. bottom level of FSH.

	Case/participants	OR (95% CI)	*p*	*p* for interaction
Age (y)				.571
<60	181/312	0.566 (0.252–1.273)	.169	
≥60	151/271	0.678 (0.297–1.544)	.354	
Diabetes duration (m)				.261
<121	181/294	0.393 (0.175–0.881)	.023	
≥121	151/289	0.960 (0.424–2.174)	.923	
Overweight/obesity				.922
No	126/294	0.453 (0.214–0.962)	.039	
Yes	206/289	1.034 (0.416–2.570)	.943	
Central obesity				.062
No	75/201	0.323 (0.116–0.902)	.031	
Yes	257/382	0.845 (0.422–1.691)	.634	
Hyperuricemia				.568
No	246/441	0.637 (0.344–1.177)	.150	
Yes	84/138	0.616 (0.128–2.961)	.545	
Hypertension				.165
No	98/207	0.692 (0.277–1.726)	.430	
Yes	234/376	0.519 (0.247–1.087)	.082	
Dyslipidemia				.414
No	96/206	0.704 (0.277–1.790)	.461	
Yes	236/377	0.561 (0.273–1.165)	.122	

*Note*: Adjusted for age, body mass index, waist circumference, diabetes duration, hypertension, hyperuricemia, glycemic control, dyslipidemia, luteinizing hormone, prolactin, and estradiol. *P* was calculated by multivariate logistic regression analyses.

Abbreviations: CI, confidence intervals; FSH, follicle‐stimulating hormone; NAFLA, nonalcoholic fatty liver disease; OR, odds ratio. [Correction added on 8 June 2023, after first online publication: in table 4, column 1, ‘Central obesity’ has been updated to ‘Hyperuricemia’.]

## 4 DISCUSSION

This study revealed that serum FSH levels were negatively associated with the prevalence of NAFLD in postmenopausal women with T2DM, even after adjusting for demographic, metabolic indexes, and other related hormones levels.

NAFLD was thought to be a manifestation of metabolic syndrome (MetS) in the liver. Previous studies had explored that high serum FSH level was inversely associated with metabolic disease in postmenopausal women. For example, Zhang et al found that a lower serum FSH level was associated with higher fasting blood glucose, TC, BP, and BMI and lower HDL‐c in postmenopausal women.^24^ Chu et al found that premenopausal women with higher basal FSH levels (FSH >7 IU/L) had significantly higher TC and LDL. They found it appears earlier than E2 deficiency in postmenopausal women, and the higher FSH the higher LDL.^25^ In addition, Wang et al also found that low serum FSH level was associated with higher prevalence of prediabetes, diabetes, and NAFLD and higher cardiovascular disease risk in postmenopausal women.^18^, ^26^, ^27^ FSH may be a better indicator of the occurrence of MetS than C‐reactive protein (CRP) and leptin, and the diagnostic value of FSH for MetS appeared to be similar to adiponectin and leptin‐to‐adiponectin ratio.^15^ Thus, we hypothesized that high serum FSH level could decrease the prevalence of NAFLD by improving MetS. In our study, we also found that, in postmenopausal women with T2DM, high serum FSH level was associated with lower BMI, WC, and the prevalence of NAFLD, which was thought to be a manifestation of MetS in the liver. This result was consistent with previous studies.

The mechanism of the association between FSH and NAFLD remains unclear. Insulin resistance was thought to partially explain the negative association between FSH and NAFLD. When women went into postmenopause, FSH concentration was regulated by follistatin and activin A, in addition to the hypothalamic–pituitary‐gonadal axis.^28^ An increase in FSH was accompanied by a decrease in follistatin and an increase in activin A.^29^ An in vitro study discovered that activin A played anti‐inflammatory and antioxidant roles in endothelial cells and insulin sensitization and anti‐inflammatory roles in human islet cells, which demonstrated that activin A could prevent hyperglycemia, hyperinsulinemia, and inflammation.^28^, ^30^ In addition, Bruning et al found that when the central nervous system insulin receptor gene was knocked out, insulin resistance and hypogonadatropism were developed in both male and female mice, leading to the decrease of LH and FSH concentrations.^31^ Thus, with the increase of FSH levels, individuals had less insulin resistance, which further decreased the prevalence of NAFLD. In our study, we came to similar results. We calculated HOMA‐IR to assess insulin resistance. We found that FSH was negatively associated with HOMA‐IR (Table 3).

The negative association between FSH and NAFLD could also be explained by obesity. When women reached postmenopause, adipose tissue became an important source of estradiol. Aromatase in adipose tissue could convert androgens into estrogens.^32^ Thus, compared with nonobese person, postmenopausal women with obesity had higher estradiol, which further contributed to lower FSH. In addition, Zhang et al found that weight loss could induce the elevation of FSH level.^33^ Sowers found that the change in logFSH was positively correlated with the change in log (fat mass).^34^ We hypothesized that, in postmenopausal women, as FSH levels rose, so did fat mass, resulting in increased production of endogenous estrogen, which in turn reduced FSH levels. Therefore, in our study, we found the higher the FSH level, the lower the BMI and the lower prevalence of NAFLD.

However, the basic research showed different results. Song et al confirmed the existence of FSH receptor (FSHR) in liver tissue. They found that FSH binds to its specific high‐affinity receptor FSHR and inhibits the expression of LDL receptor (LDLR) in a concentration‐dependent manner, thereby inhibiting LDL metabolism.^35^ Studies in mice also found that FSH could promote lipid synthesis and lipid drome formation in visceral fat through the Gαi/Ca2+/CREB pathway,^36^ and activate endothelial VACM‐1through the FSHR/Gas/cAMP/PKA and PI3K/Akt/mTOR/NF‐kB signaling pathways to promote atherosclerosis.^37^ Blocking FSH could inhibit liver cholesterol biosynthesis and reduce serum cholesterol content.^38^ The reason the results of basic research were different from ours might be that the mice selected for basic research were standard mice, whose FSH levels had been changed by ovariectomy. FSH level was artificially modified and was not affected by other factors. However, postmenopausal women had increased fat mass, which increased the production of endogenous estrogen and lower FSH level. FSH level varied depending on the condition of the body. Thus, the potential mechanism of FSH and NAFLD needs further exploration.

Our study has some strengths. First, we analyzed the association between FSH and NAFLD in a population with diabetes, which could exclude the possible influence of diabetes. Second, we analyzed the relationship between FSH and NAFLD in different subgroups, further proving that FSH was an independent influence factor of NAFLD. However, there are still some limitations to our study. First, the study is a cross‐sectional study, so we are unable to determine the causal relationship between FSH and NAFLD. Second, the study population is hospitalized patients with diabetes, so this conclusion cannot be extended to the general population. Third, we define menopause by retrospective questionnaires and there may be some recall error and some artificial menopause cannot be completely avoided. And we measured FSH only once, but this did not have a significant impact on the results because most of the subjects we included were in the postmenopausal period, when FSH levels were relatively stable. Finally, the diagnosis of NAFLD by liver ultrasound has some limitations, but as the gold standard for diagnosis, liver puncture is an invasive examination, which is not widely applied in clinical practice. We will conduct further research to confirm it.

In conclusion, serum FSH level was negatively associated with the prevalence of NAFLD in postmenopausal women with T2DM. This relationship did not disappear with changes in other metabolic disease such as obesity, hypertension, and dyslipidemia. FSH might be a potential index for screening and identifying individuals with high risk of NAFLD in postmenopausal women. Further studies are needed to explore more relevant mechanism between FSH and NAFLD.

## CONFLICT OF INTEREST STATEMENT

The authors declare that they have no conflicts of interest.
